# First-line treatment of camrelizumab combined with chemotherapy in advanced gastroenteropancreatic neuroendocrine carcinoma: Study protocol for a prospective, multicenter, phase II study

**DOI:** 10.3389/fonc.2022.958905

**Published:** 2022-09-16

**Authors:** Xiaofen Li, Qing Ma, Chen Chang, Hao Li, Dan Cao

**Affiliations:** ^1^ Department of Abdominal Oncology, West China Hospital, Sichuan University, Chengdu, China; ^2^ General Practice Ward/International Medical Center Ward, West China Hospital, Sichuan University/West China School of Nursing, Sichuan University, Chengdu, China; ^3^ West China School of Medicine, Sichuan University, Chengdu, China

**Keywords:** gastroenteropancreatic neuroendocrine carcinoma, camrelizumab, immunotherapy, first line, study protocol

## Abstract

**Background:**

Gastroenteropancreatic neuroendocrine carcinoma (GEP-NEC) is a group of rare but highly aggressive malignancies. The standard chemotherapy regimens composed of etoposide and cisplatin/carboplatin (EP/EC) are of limited efficacy. This prospective, multicenter, phase II study is conducted to explore the effectiveness and safety of first-line anti-PD-1 antibody (camrelizumab) combined with chemotherapy in advanced GEP-NEC patients.

**Methods:**

Patients with unresectable or metastatic GEP-NEC will receive camrelizumab combined with standard first-line chemotherapy every 3 weeks (camrelizumab 200 mg, administered intravenously on day 1; etoposide 100 mg/m^2^, administered intravenously on days 1–3; cisplatin 75 mg/m^2^, administered intravenously on day 1 or carboplatin area under the curve 5 mg/ml per min, administered intravenously on day 1). All patients were naïve to systemic therapy in the advanced setting. The primary endpoint is a 6-month progression-free survival (PFS) rate. The secondary endpoints are objective response rate, PFS, overall survival and adverse reactions.

**Discussion:**

This is the first study to investigate the therapeutic potential of camrelizumab plus chemotherapy for advanced GEP-NEC. It is expected that this trial will propose a new and effective treatment strategy for GEP-NEC in the first-line setting.

**Clinical Trial Registration:**

This trial is registered at the Chinese Clinical Trial Registry http://www.chictr.org.cn, identifier ChiCTR2100047314.

**Date of Registration:**

June 12, 2021.

## Background

According to the 2019 WHO Classification of Tumors of Digestive System, gastroenteropancreatic neuroendocrine neoplasms (GEP-NENs) are classified into well-differentiated neuroendocrine tumors (NETs), poorly differentiated neuroendocrine carcinomas (NECs), and mixed neuroendocrine–non-neuroendocrine neoplasms (1, 2). GEP-NECs are composed of highly atypical cells with high proliferative activity (>20 mitoses/2 mm^2^ or a Ki-67 index >20%) and poor prognosis. In recent decades, the incidence of GEP-NEC has been reported to have increased rapidly (3, 4).

Despite progressing understanding of NECs and emerging new therapies, the standard first-line treatment for GEP-NEC patients is still platinum-based chemotherapy, which has been used for over 30 years (5, 6). However, the efficacy and long-lasting response of platinum-based chemotherapy (etoposide and cisplatin/carboplatin, EP/EC regimens) is very limited. The reported objective response rate (ORR) ranges from 20% to 40%, with a median progression-free survival (PFS) of 2–6 months and an overall survival (OS) of 6–12 months (7). In the NORDIC NEC study, investigators retrospectively analyzed 305 patients with advanced gastrointestinal NEC and found that the ORR of the first-line platinum-based chemotherapy was 31%, with a median PFS of 4 months (95% confidence interval (CI) 3.1–4.6 months) and a median OS of 11 months (95% CI 9.4–12.6 months) (8). In fact, recommendations of the first-line platinum-based chemotherapy for GEP-NECs are mainly based on non-randomized, small sample size, and retrospective studies. Further prospective studies with new treatment approaches are in urgent need for GEP-NEC patients.

Recently, immune checkpoint inhibitors (ICIs) have shown remarkable responses in various solid malignancies, especially in tumors with a high mutational rate, deficient mismatch repair (dMMR) status, or positive PD-L1 expression. Preclinical studies have shown a high tumor mutational rate (TMB) and high frequency of positive PD-L1 expression in neuroendocrine carcinomas, which indicates immunogenic and promising responsiveness to ICIs (9–11). In small cell neuroendocrine carcinoma of the lung, which is similar to GEP-NEC in pathology, two randomized, controlled, phase 3 trials have demonstrated the survival benefits of ICIs (atezolizumab and durvalumab) combined with EP/EC chemotherapy (12, 13). Thus, atezolizumab or durvalumab combined with chemotherapy has been recommended as the standard first-line treatment for small cell lung cancer by the National Comprehensive Cancer Network (NCCN) guidelines (14). Additionally, some small size trials have revealed the efficacy and manageable toxicity of ICIs in refractory high-grade NENs (15–18). In the DART SWOG 1609 study, nivolumab plus ipilimumab showed an ORR of 44% and a 6-month PFS rate of 44% in refractory high-grade gastrointestinal and pulmonary NECs (15). A recently published study showed an encouraging response of sintilimab in refractory GEP-NECs with an ORR of 27.8% (18).

Inspired by preclinical work and immunotherapy in small cell lung cancer, we conducted this study to explore the benefit of an anti-PD-1 antibody, camrelizumab, combined with chemotherapy as first-line treatment in advanced GEP-NEC patients.

## Methods and analysis

### Study design and treatment

This prospective, multicenter, single-arm, phase II study is designed to investigate the efficacy and safety of first-line camrelizumab combined with standard chemotherapy in patients diagnosed with advanced GEP-NEC. The study protocol is formulated in accordance with the SPIRIT (Standard Protocol Items: Recommendations for Interventional Trials) statement (19). The trial has obtained ethics approval by the Chinese Ethics Committee of Registering Clinical Trials and is currently ongoing in six medical facilities in China. This protocol is version 3.0 revised on 21 January 2022.

The main inclusion criteria are pathologically confirmed locally advanced or metastatic extra-pulmonary neuroendocrine carcinoma (EP-NEC), with the primary sites located in non-pulmonary organs such as the gastrointestinal tract, pancreas, or biliary tract; without previous first-line systemic antitumor therapy since advanced GEP-NEC diagnosis; at least a 6-month interval between cancer recurrence or metastasis and the end of adjuvant chemotherapy for patients who received prior radical surgery and adjuvant chemotherapy; aged 18 to 75 years; life expectancy of over 3 months; ECOG performance status 0 or 1; at least one measurable lesion according to the Response Evaluation Criteria in Solid Tumors (RECIST) version 1.1 (20); and adequate organ function. **Table 1** lists complete inclusion and exclusion criteria.

**Table 1:** Patient inclusion and exclusion criteria.

Inclusion criteria	Exclusion criteria
1. Histologically and/or cytologically confirmed, unresectable locally advanced or metastatic extrapulmonary neuroendocrine carcinoma (EP-NEC), with primary sites located in nonpulmonary organs such as the gastrointestinal tract, pancreas, or biliary tract;The pathological diagnosis of gastrointestinal neuroendocrine carcinoma will be established according to the 2019 WHO Classification of Tumours of the Digestive System; 2. Age from 18 to 75 years, either sex; 3. Life expectancy of ≥3 months; 4. Eastern Cooperative Oncology Group (ECOG) performance status score 0~1; 5. No prior first-line systemic antitumor therapy since advanced GEP-NEC diagnosis; at least a 6-month interval between cancer recurrence or metastasis and the end of adjuvant chemotherapy for patients received prior radical surgery and adjuvant chemotherapy; 6. Measurable disease according to RECIST criteria version 1.1. 7. Adequate hematologic, hepatic, and renal functions: hemoglobin ≥90 g/l, neutrophils ≥1,500/mm^3^, platelets ≥75,000/mm^3^; aspartate aminotransferase and alanine aminotransferase ≤3.0 × upper limit of normal (ULN), or ≤5.0 × ULN in case of liver metastasis; bilirubin ≤1.5 × ULN; creatinine ≤1.5 × ULN, creatinine clearance ≥50 ml/min; activated partial thromboplastin time, prothrombin time and international normalized ratio ≤1.5 × ULN; 8. In case of active hepatitis B or C, antiviral therapy starting at least 14 days before experimental drug administration and HBV DNA ≤2,500 copies/mL or ≤500IU/ml and HCV RNA within the lower limit of detection; 9. Informed consent form signed.	1. Histologically confirmed well-differentiated neuroendocrine tumor, mixed adenoneuroendocrine carcinoma, etc.; 2. Severe hepatic or renal insufficiency; 3. Myocardial infarction within 3 months; 4. Other malignancy history with disease free survival <5 years, except for curative *in situ* cervical cancer, curative skin basal cell carcinoma and curative gastrointestinal cancer by endoscopic mucoresection; 5. Current or past history of autoimmune diseases, including but not limited to: interstitial lung disease, uveitis, enteritis, nephritis, hyperthyroidism, and hypothyroidism; 6. Active pulmonary tuberculosis within 1 year; 7. Severe and uncontrolled internal medicine diseases; 8. Severe infection needing intravenous antibiotics, antifungal agents, antiviral drugs, etc.; 9. Pregnant or breastfeeding woman; man and woman unwilling to take any contraceptive measures; 10. Long-term history of chronic diarrhea, or complete intestinal obstruction; 11. Immunosuppressant or corticosteroid (systemic or local) use to suppress immune function within 2 weeks before inclusion; 12. Ever received treatment of immune checkpoint inhibitors; 13. Allergic disease history, severe hypersensitivity to experimental drugs; 14. Congenital or acquired immunodeficiency such as HIV infection; 15. Other conditions that investigators consider not suitable for this study.

**Figure 1:**
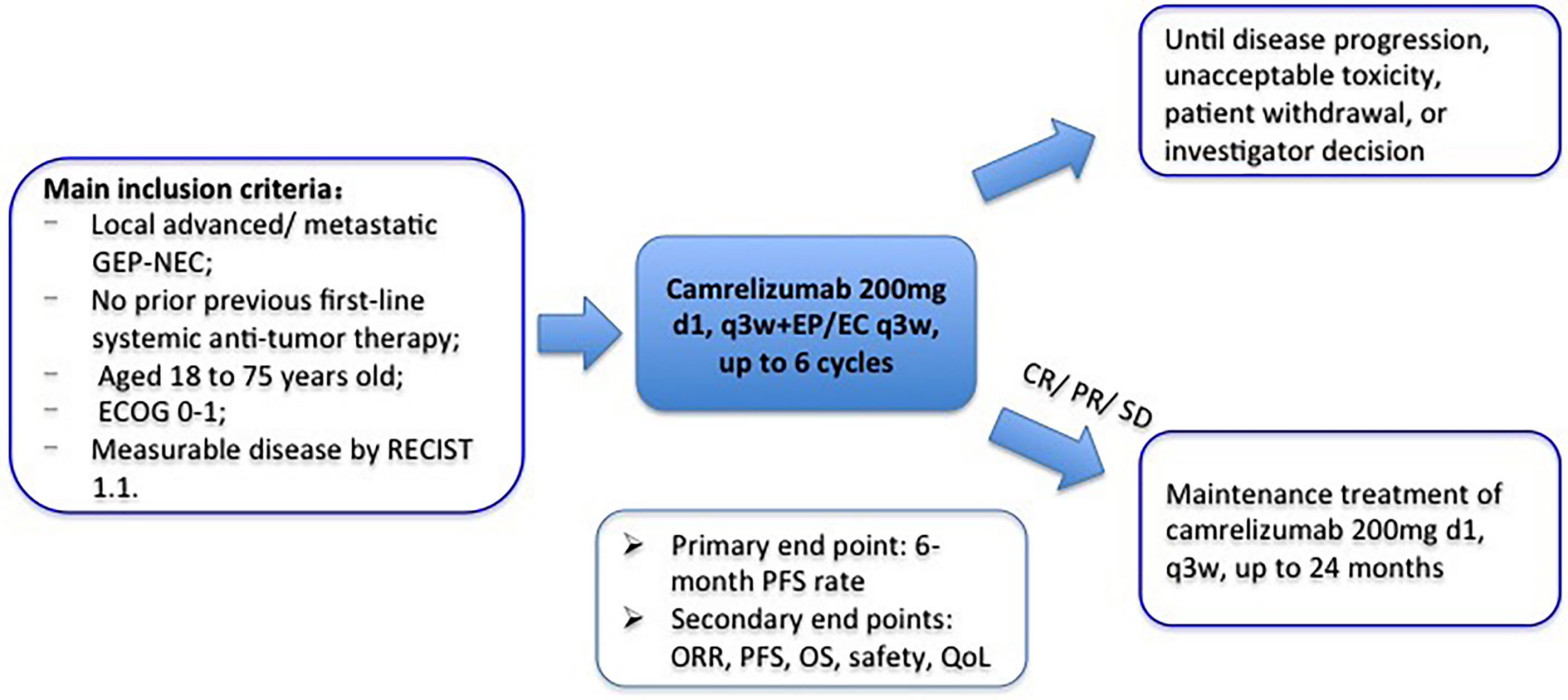
Outline of the study design.

Patients eligible for participation and having signed informed consents will receive camrelizumab and EP/EC chemotherapy every 3 weeks (camrelizumab 200 mg ivgtt on day 1 + etoposide 100 mg/m^2^ ivgtt on days 1–3 + cisplatin 75 mg/m^2^ ivgtt on day 1 or carboplatin area under the curve 5 mg/ml per min, ivgtt on day 1). The total dose of cisplatin can be administered in 3 days. Treatment continues until disease progression, intolerable toxicity, patient refusal, or investigator decision. Patients will receive maintenance treatment of camrelizumab (200 mg, q3w) if disease stable or remission after 6 cycles of combination therapy. The treatment duration of camrelizumab is at most 24 months. The study design is shown in **Figure 1**.

### Endpoints and assessments

The primary endpoint of this trial is the 6-month PFS rate. The secondary endpoints are ORR, PFS, OS, safety, and quality of life. The disease evaluation is performed every 9 weeks by computed tomography (CT) based on RECIST version 1.1. Patients who get radiological disease progression (PD) could be permitted to continue treatment if investigators judge the clinical benefit from the continued treatment (according to physical status, clinical symptoms, and laboratory examination results). Moreover, these patients should receive imaging examination again to determine whether they get confirmed PD after 4 weeks according to immune-related RECIST (irRECIST) (21). PFS is defined as the time from trial enrollment to first confirmed PD or death. OS is defined as the time from trial enrollment to death of any cause. And ORR is defined as the percentage of patients that obtain complete response (CR) or partial response (PR).

Serum and tissue samples will be collected before treatment start for cytokines, TMB, microsatellite instability, and PD-L1 analyses. All adverse events will be monitored during the treatment period, which will be assessed according to the Common Terminology Criteria for Adverse Events (CTCAE) version 5.0. The quality of life will be evaluated at baseline and prior to every imaging evaluation, using the validated European Organization for Research and Treatment of Cancer Quality of Life Core Questionnaire 30 (EORTC QLQ-C30) (22).

### Sample size and statistical analyses

This study aimed to achieve a 6-month PFS rate of 50%. Sample size was determined using Simon’s optimal two-stage design for the primary endpoint of 6-month PFS rate (revised in version 4.0 of the protocol with approval from the ethics committee), with a type I error of 0.05 and 80% power. In the first stage, 6 evaluable patients were enrolled; if at least 2 patients achieved a PFS of ≥ 6 months, the study would proceed to the second stage with an additional 14 patients, for a total of 20 evaluable patients. The regimen would be considered promising if at least 8 patients in total achieved a 6-month PFS or longer. Assuming an approximately 10% non-evaluable/ dropout rate, we planned to enroll about 22 patients to obtain 20 evaluable patients.

## Discussion

GEP-NECs are characterized by rapid deterioration and poor prognosis. The current recommended first-line platinum-based chemotherapy is of limited efficacy. A new treatment strategy is in urgent need for GEP-NECs.

As aforementioned, studies have revealed that NECs have features of high TMB and PD-L1-positive expression, indicating immunogenic and promising responsiveness to immune checkpoint inhibitors (9–11). In addition, immune checkpoint inhibitors have significant survival benefit in small cell lung cancer (12, 13) and Merkel cell carcinoma (23, 24), both of which are with similar biological characteristics to GEP-NEC. Inspired by these results, we have planned this trial to investigate the efficacy and safety of first-line immunotherapy combined with standard platinum-based chemotherapy in advanced GEP-NEC patients.

At present, there are a few studies investigating the value of immunotherapy in refractory advanced gastrointestinal NECs (15, 16). The phase II study, DART SWOG 1609, explored the efficacy of nivolumab plus ipilimumab in refractory non-pancreatic neuroendocrine tumors. The results showed a relatively high ORR of 44% (8/18) and acceptable toxicity in patients with gastrointestinal and pulmonary NECs (15). However, to date, there is no published study exploring the benefit of first-line immunotherapy plus chemotherapy in GEP-NECs. We believe our study will propose a brand new treatment approach for advanced GEP-NECs.

There are several limitations in our study, including lack of a control group, a small sample size, and uncertain predictive biomarkers of therapeutic effect. Besides, as this is an investigator-initiated trial with limited budgets, the histological and radiological evaluations are performed in local centers without centralized assessment.

## Data availability statement

The original contributions presented in the study are included in the article/Supplementary Material. Further inquiries can be directed to the corresponding author.

## Ethics statement

This study was reviewed and approved by Chinese Ethics Committee of Registering Clinical Trials. The patients/participants provided their written informed consent to participate in this study.

## Author contributions

DC and XL are responsible for the trial design. XL is responsible for recruitment and patient information. QM and CC are responsible for patient follow-up. QM and HL are responsible for data collection. XL and CC are responsible for statistical analysis. XL, QM, and DC drafted and revised the manuscript. All authors contributed to the article and approved the submitted version.

## Funding

This study is supported by the Sichuan Province Science and Technology Support Program (2021YFS0047). The main role of the funding is providing financial support for this study.

## Acknowledgments

We gratefully acknowledge the understanding and cooperation of enrolled patients.

## Conflict of interest

The authors declare that the research was conducted in the absence of any commercial or financial relationships that could be construed as a potential conflict of interest.

## Correction note

A correction has been made to this article. Details can be found at: 10.3389/fonc.2026.1726750.

## Publisher’s note

All claims expressed in this article are solely those of the authors and do not necessarily represent those of their affiliated organizations, or those of the publisher, the editors and the reviewers. Any product that may be evaluated in this article, or claim that may be made by its manufacturer, is not guaranteed or endorsed by the publisher.

## References

[1] WHO . WHO classification of tumours editorial board. In: WHO classification of tumours of digestive system. Lyon: IARC Press (2019).

[2] Nagtegaal ID Odze RD Klimstra D Paradis V Rugge M Schirmacher P . The 2019 WHO classification of tumours of the digestive system. Histopathology (2020) 76(2):182–8. doi: 10.1111/his.13975, PMID: 31433515 PMC7003895

[3] Dasari A Shen C Halperin D Zhao B Zhou S Xu Y . Trends in the incidence, prevalence, and survival outcomes in patients with neuroendocrine tumors in the united states. JAMA Oncol (2017) 3(10):1335–42. doi: 10.1001/jamaoncol.2017.0589, PMID: 28448665 PMC5824320

[4] Fraenkel M Kim M Faggiano A de Herder WW Valk GD NETwork K . Incidence of gastroenteropancreatic neuroendocrine tumours: a systematic review of the literature. Endocrine-Related Cancer (2014) 21(3):R153–63. doi: 10.1530/ERC-13-0125, PMID: 24322304

[5] Pavel M Oberg K Falconi M Krenning EP Sundin A Perren A . Gastroenteropancreatic neuroendocrine neoplasms: ESMO clinical practice guidelines for diagnosis, treatment and follow-up. Ann Oncol (2020) 31(7):844–60. doi: 10.1016/j.annonc.2020.03.304, PMID: 32272208

[6] Garcia-Carbonero R Sorbye H Baudin E Raymond E Wiedenmann B Niederle B . ENETS consensus guidelines for high-grade gastroenteropancreatic neuroendocrine tumors and neuroendocrine carcinomas. Neuroendocrinology (2016) 103(2):186–94. doi: 10.1159/000443172, PMID: 26731334

[7] Mollazadegan K Welin S Crona J . Systemic treatment of gastroenteropancreatic neuroendocrine carcinoma. Curr Treat Option Oncol (2021) 22(8):68. doi: 10.1007/s11864-021-00866-9, PMID: 34110508 PMC8192386

[8] Sorbye H Welin S Langer SW Vestermark LW Holt N Osterlund P . Predictive and prognostic factors for treatment and survival in 305 patients with advanced gastrointestinal neuroendocrine carcinoma (WHO G3): the NORDIC NEC study. Ann Oncol (2013) 24(1):152–60. doi: 10.1093/annonc/mds276, PMID: 22967994

[9] Peifer M Fernandez-Cuesta L Sos ML George J Seidel D Kasper LH . Integrative genome analyses identify key somatic driver mutations of small-cell lung cancer. Nat Genet (2012) 44(10):1104–10. doi: 10.1038/ng.2396, PMID: 22941188 PMC4915822

[10] Vijayvergia N Boland PM Handorf E Gustafson KS Gong YL Cooper HS . Molecular profiling of neuroendocrine malignancies to identify prognostic and therapeutic markers: a fox chase cancer center pilot study. Br J Cancer (2016) 115(5):564–70. doi: 10.1038/bjc.2016.229, PMID: 27482646 PMC4997552

[11] Kim ST Ha SY Lee S Ahn S Lee J Park SH . The impact of PD-L1 expression in patients with metastatic GEP-NETs. J Cancer (2016) 7(5):484–9. doi: 10.7150/jca.13711, PMID: 26958083 PMC4780123

[12] Horn L Mansfield AS Szczesna A Havel L Krzakowski M Hochmair MJ . First-line atezolizumab plus chemotherapy in extensive-stage small-cell lung cancer. N Engl J Med (2018) 379(23):2220–9. doi: 10.1056/NEJMoa1809064, PMID: 30280641

[13] Paz-Ares L Dvorkin M Chen Y Reinmuth N Hotta K Trukhin D . Durvalumab plus platinum–etoposide versus platinum–etoposide in first-line treatment of extensive-stage small-cell lung cancer (CASPIAN): a randomised, controlled, open-label, phase 3 trial. Lancet (2019) 394(10212):1929–39. doi: 10.1016/S0140-6736(19)32222-6, PMID: 31590988

[14] Ganti AKP Loo BW Bassetti M Blakely C Chiang A D'Amico TA . Small cell lung cancer, version 2.2022, NCCN clinical practice guidelines in oncology. J Natl Compr Canc Netw (2021) 19(12):1441–64. doi: 10.6004/jnccn.2021.0058, PMID: 34902832 PMC10203822

[15] Patel SP Othus M Chae YK Giles FJ Hansel DE Singh PP . A phase II basket trial of dual anti-CTLA-4 and anti-PD-1 blockade in rare tumors (DART SWOG 1609) in patients with nonpancreatic neuroendocrine tumors. Clin Cancer Res (2020) 26(10):2290–6. doi: 10.1158/1078-0432.CCR-19-3356, PMID: 31969335 PMC7231627

[16] Strosberg J Mizuno N Doi T Grande E Delord JP Shapira-Frommer R . Efficacy and safety of pembrolizumab in previously treated advanced neuroendocrine tumors: Results from the phase II KEYNOTE-158 study. Clin Cancer Res (2020) 26(9):2124–30. doi: 10.1158/1078-0432.CCR-19-3014, PMID: 31980466 PMC7811789

[17] Mehnert JM Bergsland E O'Neil BH Santoro A Schellens JHM Cohen RB . Pembrolizumab for the treatment of programmed death-ligand 1-positive advanced carcinoid or pancreatic neuroendocrine tumors: Results from the KEYNOTE-028 study. Cancer (2020) 126(13):3021–30. doi: 10.1002/cncr.32883, PMID: 32320048

[18] Jia R Li Y Xu N Jiang HP Zhao CH Liu RR . Sintilimab in patients with previously treated metastatic neuroendocrine neoplasms. Oncol (2022) 27:e625–32. doi: 10.1093/oncolo/oyac097, PMID: 35647908 PMC9355821

[19] Chan AW Tetzlaff JM Altman DG Laupacis A Gotzsche PC Krleza-Jeric K . SPIRIT 2013 statement: defining standard protocol items for clinical trials. Ann Intern Med (2013) 158(3):200–7. doi: 10.7326/0003-4819-158-3-201302050-00583, PMID: 23295957 PMC5114123

[20] Eisenhauer EA Therasse P Bogaerts J Schwartz LH Sargent D Ford R . New response evaluation criteria in solid tumours: Revised RECIST guideline (version 1.1). Eur J Cancer (2009) 45(2):228–47. doi: 10.1016/j.ejca.2008.10.026, PMID: 19097774

[21] Nishino M Giobbie-Hurder A Gargano M Suda M Ramaiya NH Hodi FS . Developing a common language for tumor response to immunotherapy: Immune-related response criteria using unidimensional measurements. Clin Cancer Res (2013) 19(14):3936–43. doi: 10.1158/1078-0432.CCR-13-0895, PMID: 23743568 PMC3740724

[22] Aaronson NK Ahmedzai S Bergman B Bullinger M Cull A Duez NJ . The European-Organization-for-Research-and-Treatment-of-Cancer qlq-C30 - a quality-of-Life instrument for use in international clinical-trials in oncology. J Natl Cancer Institute (1993) 85(5):365–76. doi: 10.1093/jnci/85.5.365, PMID: 8433390

[23] Nghiem PT Bhatia S Lipson EJ Kudchadkar RR Miller NJ Annamalai L . PD-1 blockade with pembrolizumab in advanced merkel-cell carcinoma. N Engl J Med (2016) 374(26):2542–52. doi: 10.1056/NEJMoa1603702, PMID: 27093365 PMC4927341

[24] D’Angelo SP Russell J Lebbe C Chmielowski B Gambichler T Grob JJ . Efficacy and safety of first-line avelumab treatment in patients with stage IV metastatic merkel cell carcinoma: A preplanned interim analysis of a clinical trial. JAMA Oncol (2018) 4(9):e180077. doi: 10.1001/jamaoncol.2018.0077, PMID: 29566106 PMC5885245

