# Correction: First-line treatment of camrelizumab combined with chemotherapy in advanced gastroenteropancreatic neuroendocrine carcinoma: study protocol for a prospective, multicenter, phase II study

**DOI:** 10.3389/fonc.2026.1726750

**Published:** 2026-03-02

**Authors:** Xiaofen Li, Qing Ma, Chen Chang, Hao Li, Dan Cao

**Affiliations:** 1 Department of Abdominal Oncology, West China Hospital, Sichuan University, Chengdu, China; 2 General Practice Ward/International Medical Center Ward, West China Hospital, Sichuan University/West China School of Nursing, Sichuan University, Chengdu, China; 3 West China School of Medicine, Sichuan University, Chengdu, China

**Keywords:** gastroenteropancreatic neuroendocrine carcinoma, camrelizumab, immunotherapy, first line, study protocol

The sample size calculation was updated based on a revised statistical design.

A correction has been made to the section **Methods and analysis**, *Sample size and statistical analyses*, Paragraph 1:

“This study aimed to achieve a 6-month PFS rate of 50%. Sample size was determined using Simon’s optimal two-stage design for the primary endpoint of 6-month PFS rate (revised in version 4.0 of the protocol with approval from the ethics committee), with a type I error of 0.05 and 80% power. In the first stage, 6 evaluable patients were enrolled; if at least 2 patients achieved a PFS of ≥ 6 months, the study would proceed to the second stage with an additional 14 patients, for a total of 20 evaluable patients. The regimen would be considered promising if at least 8 patients in total achieved a 6-month PFS or longer. Assuming an approximately 10% non-evaluable/ dropout rate, we planned to enroll about 22 patients to obtain 20 evaluable patients.”

The tumor type was updated. A correction has been made to the section **Methods and analysis**, *Study design and Treatment*, Paragraph 2:

“The main inclusion criteria are pathologically confirmed locally advanced or metastatic extra-pulmonary neuroendocrine carcinoma (EP-NEC), with the primary sites located in non-pulmonary organs such as the gastrointestinal tract, pancreas, or biliary tract;”

Also revised in **Table 1** inclusion 1:

“1. Histologically and/or cytologically confirmed, unresectable locally advanced or metastatic extrapulmonary neuroendocrine carcinoma (EP-NEC), with primary sites located in non-pulmonary organs such as the gastrointestinal tract, pancreas, or biliary tract;The pathological diagnosis of gastrointestinal neuroendocrine carcinoma will be established according to the 2019 WHO Classification of Tumours of the Digestive System;”

The original version of this article has been updated.

